# Getting to know our non-traditional and rejected medical school applicants

**DOI:** 10.1007/s40037-020-00579-z

**Published:** 2020-04-08

**Authors:** Anouk Wouters

**Affiliations:** Faculty of Medicine Vrije Universiteit Amsterdam, Research in Education, Amsterdam UMC, Amsterdam, The Netherlands

Designing admissions policies to meet academic and non-academic selection aims while ensuring fair access proves challenging [1, 2]. Cleland et al. have previously described medical school admissions as a wicked problem, recognizing its complexity, describing selection as dynamic rather than a static solution [3]. Admissions policies are adjusted continuously in response to evaluations of their effectiveness as well as unintended consequences, such as effects on equity. The struggle between a meritocratic approach and the pursuit of equal opportunities is reflected in the rise of widening access criteria and widening participation initiatives to mitigate the effects of admissions policies on student diversity.

Over the past years, concerns about inequalities in admissions have yielded an increase in research investigating the mechanisms through which non-traditional students (e.g., first generation higher education students, students from low socioeconomic backgrounds, etc.) might be disadvantaged and their aspirations affected by admissions practices. In this issue, Ball and colleagues contribute to our understanding of the experiences of these students. They observe that non-traditional applicants use social comparison to assess their suitability for the medical study and chances of success in the admissions process [4]. Their paper contributes to our understanding of the importance of role models. Furthermore, it raises questions about the effects being rejected may have on all applicants, both from non-traditional and traditional backgrounds.

Previous research has shown the importance of role models, especially for non-traditional applicants who usually lack representation of people ‘like them’ in medical education and the medical profession [5–9]. The paper by Ball et al. brings to light *how* role models contribute to these applicants’ aspirations and confidence to apply to medical study. In addition, it reflects how non-traditional applicants’ limited sources of the different types of capital as described by Bourdieu (*social capital*—e.g. connections and networks; *cultural capital*—assets, e.g. competencies, skills, qualifications; *symbolic capital*—reputation and prestige, thus strongly dependent on how others see an individual; and *economic capital*—an individual’s wealth) can influence their confidence to apply [10]. A lack of medical connections, especially with similar backgrounds to theirs, makes it difficult for these applicants to prepare for selection [4, 6, 11] and envision themselves as being successful, all the more because they may encounter negative and discouraging reactions in their personal environment and have difficulty relying on their abilities and aspirations [4, 12]. Furthermore, these applicants have to draw upon sources outside their close network to be able to understand their position with regards to the selection criteria and their competitors. While other applicants may be able to draw upon various types of capital such as cultural (knowledge of the system and how to prepare) and social capital (obtaining relevant work experiences through their network), non-traditional applicants often have difficulties navigating the admissions process and lack relatable role models as sources for such capital [13, 14]. Without expert allies it is not surprising that they strongly rely and focus on non-social ‘objective’ test data, such as exam results [4]. In many countries, however, academic achievement is known to be subject to influences from students’ capital as well (e.g., attending a private or public school). As a result, both medical schools *and* applicants themselves may underestimate their academic ability. Uncertainty about their academic ability and strong emphasis on exam results to reassure their aspirations may deter non-traditional students from applying to medical school [15]. This accumulation of differences in capital between traditional and non-traditional applicants widens the gap and poses challenges to fair and accessible medical education [11].

The importance of non-social data to applicants may also have consequences further along the admissions process. If applicants are rejected, this rejection now becomes objective information which justifies their uncertainty and may guide future decisions to reapply. Research about factors influencing applicants’ choices to reapply is scarce, but personal perseverance and resilience seem important [4, 14]. Similar to the majority of selection literature, the study by Ball et al. was limited to students who had—eventually—been successful in selection. Lack of preparation was often their explanation for being rejected in previous rounds. If this is caused by their lack of knowledge of the admissions system (due to limited social capital) rather than an issue of poor motivation or unsuitability, these non-traditional students should not be discouraged to apply again. Furthermore, how applicants cope with rejection may differ between traditional and non-traditional students, as well as the resources they need or have available to do so. Drawing upon their available capital, traditional students may be better equipped to rebuild their confidence after rejection and reapply to medical school. However, non-traditional students, having overcome more barriers throughout their educational pathway (e.g., discrimination experienced by ethnic minority students [16]), may have built resilience to deal with rejection [5]. How applicants are effected by rejection warrants further research.

It has been argued that non-traditional applicants may benefit from widening access activities which include guidance by mentors [4, 11, 14]. I advocate a longitudinal mentorship in which medical students who identify as non-traditional students themselves coach applicants. This allows for social comparison with a relatable role model, and could help these students acquire and utilize capital (e.g., building a relevant network, gaining understanding of the hidden curriculum) as they navigate both towards and through medical school [17, 18].

In conclusion, it is important to acknowledge the different admissions experiences, needs and reactions traditional and non-traditional applicants may have. In using the terms “non-traditional” and “traditional” students I do not mean to imply that these are homogenous groups. To ensure fairness and equal opportunities we need more insight into the variety of the experiences across the entire applicant population.
